# Learning curve and target-based accuracy of diode laser versus scalpel incisions in a porcine skin model: a preclinical feasibility study with undergraduate dental students

**DOI:** 10.1007/s10103-026-05011-2

**Published:** 2026-08-28

**Authors:** Dorothee Spille, Sophia Lymperopoulos, Juliane Wagner, Clemens Mohr, Felix Lorich, Tim Kalsberger, Jörg Wiltfang, Johannes Spille

**Affiliations:** 1 Department of Neurosurgery, University Medical Center Hamburg-Eppendorf, Hamburg, Germany; 2 Department of Oral and Maxillofacial Surgery, University Hospital Schleswig-Holstein, Kiel, Germany; 3 Theoretical Physics, Christian-Albrechts-University Kiel, Kiel, Germany

**Keywords:** Diode laser, Gingivectomy, Dental education, Learning curve, NASA-TLX, Laser surgery

## Abstract

Purpose: Despite the growing clinical application of diode lasers in oral surgery, laser training remains underrepresented in dental curricula. This study evaluated the feasibility of structured preclinical diode-laser training and characterized the early learning curve of undergraduate dental students without prior laser experience, using target-based accuracy of laser incisions compared with a familiar scalpel reference condition. Methods: 22 dental students performed standardized linear incisions on porcine skin at three time points. Each session comprised five incisions (target: 10 mm length, 2 mm depth, minimal width): four diode-laser settings (1 W/pulsed, 3 W/pulsed, 1 W/continuous, 3 W/continuous; 445 nm wavelength; 320 μm fiber) and one scalpel incision. Incision morphology was assessed by micro-computed tomography. Operative time was recorded per incision, and subjective workload was evaluated using the NASA-TLX questionnaire. Results: Total operative time decreased significantly across sessions (p = 0.006). Per-method reductions were significant only for the two pulsed settings. Four of six NASA-TLX subscales improved (all p ≤ 0.013). Length accuracy improved significantly over time (main effect of time p < 0.001) with no method-time interaction and comparable, significant within-method gains for all methods. Depth accuracy showed only a small overall time effect and a significant within-method gain for a single laser setting (1 W pulsed). Incision width did not change significantly over sessions for any method. Laser incisions were consistently wider than scalpel incisions. Conclusion: Structured preclinical laser training on porcine skin is feasible and is associated with early gains in operative efficiency, perceived workload and length accuracy. These gains largely reflect general task familiarisation rather than a laser-specific learning advantage. The findings support integrating structured laser training using animal-tissue models into dental education but cannot establish clinical competence, equivalence to scalpel surgery, or transfer to intraoral patient care.

## Introduction

Lasers have become an established tool in oral and maxillofacial surgery and dental practice. Among the various laser systems, the diode laser has gained relevance for soft tissue surgery due to its compact design, ease of use, and favorable tissue interaction profile [1, 2]. Compared with conventional scalpel incisions, diode laser incisions are associated with reduced intraoperative bleeding, potentially improved wound healing, and greater patient comfort due to the simultaneous coagulation of blood vessels and lymphatics [3]. In dentistry, established applications include gingivectomy, frenectomy, implant uncovering, and coagulation of soft tissue lesions [4, 5].

Despite these advantages, the integration of dental laser training into undergraduate curricula remains limited. The postoperative outcome of laser-assisted procedures is substantially dependent on the operator’s experience with the specific laser system, including its tissue interaction, energy settings, and required hand speed [6]. Lasers are also increasingly being used for the treatment of biomaterials and in implant surgery; consequently, proficiency in their use is essential in clinical practice [7, 8]. As with many surgical skills, a structured learning curve is to be expected when novice operators are introduced to laser technology [9].

Animal tissue models have been widely used in surgical training, as they allow for standardized, reproducible practice under conditions that resemble the clinical setting without exposing patients to risk [10]. Porcine skin exhibits histological and biomechanical properties comparable to those of human tissue, making it a well-established ex vivo substrate for surgical training and device testing [11, 12]. Good training conditions facilitate the learning of new surgical procedures and can positively impact subjective workload [13].

The aim of this study was to evaluate the feasibility of a structured preclinical diode-laser training program and to document the early objective and subjective learning curve of undergraduate dental students without prior laser experience over three consecutive sessions. The primary outcome was the target-based accuracy of incision length, defined as the absolute deviation from the intended 10 mm. Secondary outcomes were target-based depth accuracy, incision width, operative time, and perceived workload. Because participants had prior scalpel training but no laser experience, the scalpel incision was included as a clinically familiar reference condition rather than as an experience-matched comparator. Assessing the mental workload of students using a novel surgical device for the first time may also provide insight into the demands of laser procedures early in the clinical career.

## Materials and methods

The study was designed as a prospective observational study with three measurement time points. A positive ethics decision was obtained from the Ethics Committee of the University of Kielprior to the commencement of the study. Informed written consent was obtained from all participants.

## Laser system

The SiroLaser Blue (Dentsply Sirona, Bensheim, Germany) is a dental diode laser featuring three selectable wavelengths: 445 nm (blue), 660 nm (red), and 970 nm (infrared). The total power range is 0–3 W. For this study, the preset program “gingivectomy” was selected, which operates at 445 nm. Energy was transmitted via sterile single-use flexible quartz glass fibers (EasyTips, 320 μm diameter). Prior to each session, the fiber tips were pre-angled at approximately 30° using the manufacturer’s bending tool to standardize the application angle. Appropriate laser safety equipment, including certified protective eyewear for all three wavelengths, was used throughout.

The four laser settings were selected to bracket clinically relevant parameters for gingival soft-tissue work within the preset gingivectomy program. Power levels of 1 W and 3 W represent the lower and a moderate output of the range typically applied for soft-tissue incision and coagulation with 445 nm diode systems. Pulsed and continuous emission modes were compared because they differ in their thermal behavior: pulsed delivery permits intermittent cooling and may reduce cumulative thermal tissue burden, whereas continuous emission delivers a constant energy density that facilitates coagulation. The factorial combination of these two power levels and two emission modes therefore reflects the range of settings a clinician would realistically encounter and allows assessment of their influence on incision quality and thermal effect.

## Tissue model

Porcine skin was used as the experimental substrate, consisting of the epidermis, dermis, and portions of the subcutis, with an approximate thickness of 0.8 cm. All specimens were obtained from a regional certified butcher, and the cold chain was maintained without interruption from procurement to experimental use. The histological and biomechanical properties of porcine skin closely resemble those of human skin and thus make it a valid model for surgical training [11].

## Study population and recruitment

Twenty-two undergraduate dental students (12 women, 10 men) with an average age of 25.9 years from the clinical semesters (7th to 10th) of the Christian-Albrechts-Universität Kiel were recruited through the Department of Oral and Maxillofacial Surgery. All participants had completed mandatory surgical training courses in oral and maxillofacial surgery, providing basic surgical competencies with the scalpel. No participant had any prior experience with laser surgery. All participants received comprehensive oral instruction and were informed about the study procedure and objectives prior to the first session. Participation was voluntary and could be withdrawn at any time without consequence.

## Study procedure

**Fig. 1:**
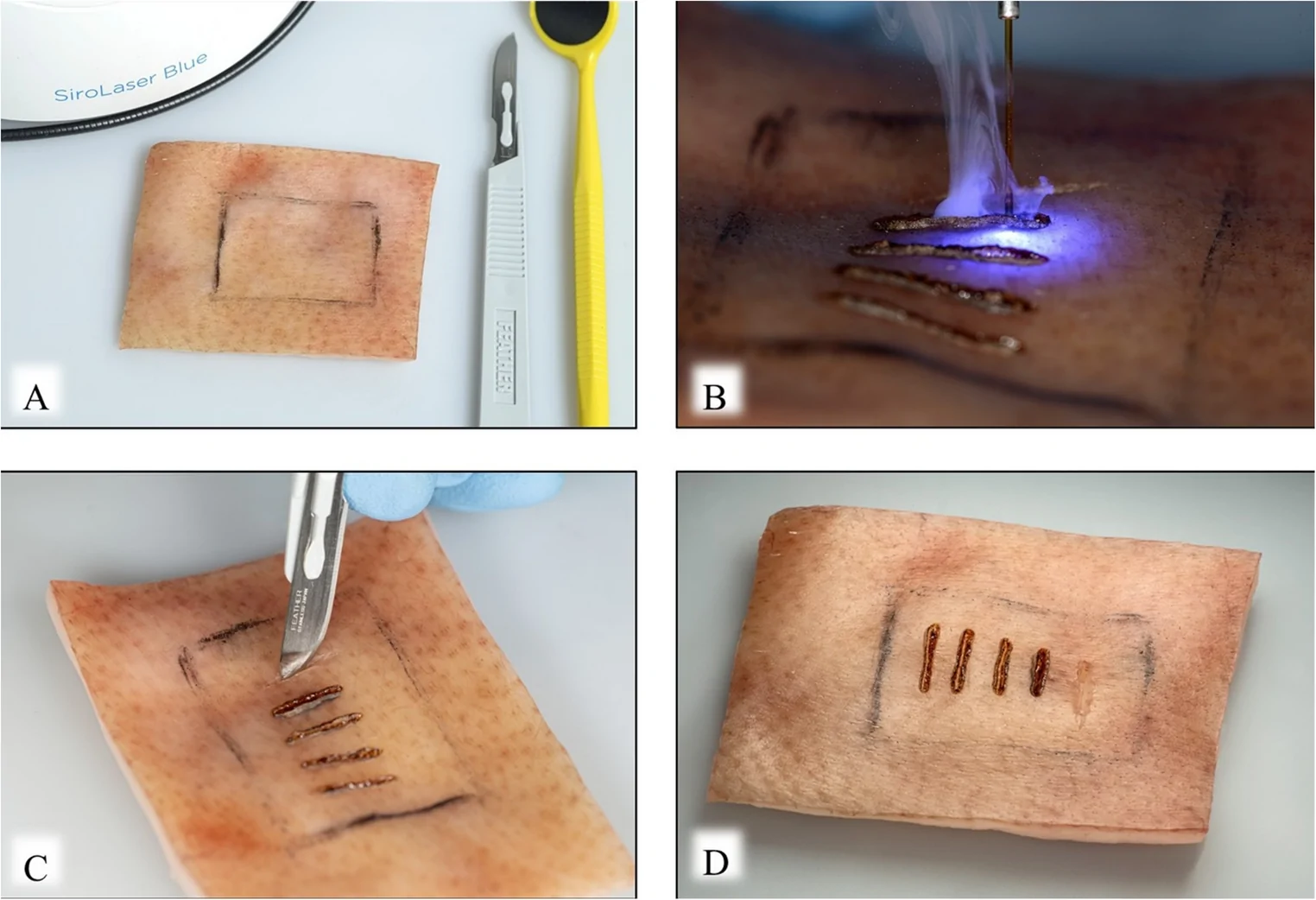
Experimental setup and procedure. (**A**) marked porcine-skin specimen (2.7 × 3.5 cm) with the SiroLaser Blue and scalpel. (**B**) laser incision in progress, showing the blue laser light and a tissue-vaporisation plume. (**C**) scalpel incision being performed. (**D**) final result showing all five incisions within the marked area

Each of the 22 participants completed three sessions at weekly intervals. No preliminary practice or test incisions were permitted. Within a predefined skin area corresponding to the dimensions of standardized histology cassettes (2.7 × 3.5 cm), five linear incisions were made per session, targeting standardized dimensions of 10 mm length, 2 mm depth, and minimal width. The five incisions were performed under the following conditions in a fixed sequence (Fig. 1): (1) laser, 1 W, pulsed mode; (2) laser, 3 W, pulsed mode; (3) laser, 1 W, continuous mode; (4) laser, 3 W, continuous mode; and (5) scalpel incision (reference). Scalpel incisions were performed using a No. 10 blade.

Following each session, participants measured the dimensions of their incision with a millimeter probe to facilitate self-reflection. Tissue specimens were excised according to the histology cassette dimensions, labeled with a color-coded thread system (T1: white; T2: violet; T3: black) attached to the upper left corner of each specimen for orientation, and fixed in 4% isotonically buffered formaldehyde solution for at least 24 h.

## Operative time

Operative time for each incision was recorded by the study supervisor using a stopwatch. The start time was defined as initial contact between the laser fiber or scalpel blade and the tissue. The end time was defined as the moment the participant verbally confirmed satisfaction with the incision result. Total operative time per session was calculated as the sum of individual incision times. In addition to the total session time, operative time was analyzed per incision method to allow method-specific efficiency to be evaluated.

### Micro-CT analysis

All formalin-fixed tissue specimens were analyzed by micro-computed tomography at the Molecular Imaging North Competence Center (MOIN CC), Christian-Albrechts-Universität Kiel. The X-ray parameters for acquiring the 3D images were set to 70 kVp, 114 µA, and 8 W, and a Cu 0.1 mm filter was employed. The resulting voxel size was 48.5 μm. The µCT allows for high-resolution, non-destructive three-dimensional documentation of incision morphology. Incision length, width, and depth were quantified from the reconstructed images using a custom Python-based surface-mapping routine and were approximated by curve fitting. A Mexican-hat function was chosen for laser incisions because their profile shows a central ablation channel flanked by raised, thermally altered margins, whereas the clean, single-troughed scalpel groove is well described by a Gaussian function. Because method-specific modeling could in principle introduce systematic measurement differences, landmark definitions were kept identical across methods. Formal intra- and inter-rater reliability of the micro-CT measurements was not assessed in the present study. This method is therefore described as feasible and potentially objective rather than formally validated.

## Histological analysis

After µCT analysis, tissue specimens were dehydrated through a graded ethanol series (50%, 70%, 80%, 90%, 100%), cleared in xylene, and embedded in paraffin. Serial sections of 3 μm thickness were cut using a sled microtome, mounted on glass slides, and dried at 37 °C. Sections were deparaffinized, rehydrated, and stained with hematoxylin-eosin (HE) according to standard protocols. HE-stained sections were examined under a transmitted-light microscope to describe tissue morphology and to identify thermally altered zones. The histological assessment was descriptive.

## NASA-TLX questionnaire

The German-validated short form of the NASA Task Load Index (NASA-TLX), translated and validated by Flägel et al. [14], was used to assess perceived workload at each session. The questionnaire comprises six subscales: mental demand, physical demand, temporal demand, goal achievement, effort, and frustration. Each subscale was rated on a Likert scale from 0 to 20, with higher values indicating greater workload.

## Statistical analysis

Descriptive statistics, including mean, standard deviation (SD), median, minimum, maximum, and 95% confidence intervals, were calculated for all continuous outcome variables. Because each participant performed all five incision conditions at each of the three time points, observations were clustered within participants and were not independent. Dimensional outcomes were therefore analyzed using a two-way repeated-measures analysis of variance (ANOVA), with incision method and time (and their interaction) as within-subject factors. The Greenhouse-Geisser correction was applied when the assumption of sphericity was violated. Within-method contrasts between time points (T1 vs. T3) were computed as paired comparisons and adjusted for multiplicity across methods using the Holm procedure. Effect sizes are reported as generalized eta-squared for omnibus effects and as paired Cohen’s d (dz) for within-method contrasts, together with 95% confidence intervals of the mean difference. Between-method comparisons at individual time points are reported with Cohen’s d as effect size.

Operative times were right-skewed and were therefore analyzed non-parametrically using the Friedman test with Holm-adjusted Wilcoxon signed-rank post-hoc comparisons, both for the total session time and for each incision method. NASA-TLX subscales were compared between T1 and T3.

Given the exploratory, feasibility-oriented design and the number of methods, dimensions, time points, and workload domains, results are interpreted as preliminary, and effect estimates with confidence intervals are emphasized alongside p-values. Analyses were performed using SPSS Statistics 31.0 (IBM, Armonk, NY, USA). A significance level of α = 0.05 was applied throughout.

## Results

### Participants

A total of 22 undergraduate dental students completed all three sessions. All participants had basic surgical training but no prior exposure to laser surgery. No dropouts occurred during the study period.

### Operative time

Total operative time per session decreased progressively across the three sessions. The mean total time was 215 s (SD 120) at T1, 173 s (SD 74.5) at T2, and 140 s (SD 62.5) at T3. The Friedman test indicated a significant effect of time, and Holm-adjusted Wilcoxon post-hoc comparisons confirmed significant reductions from T1 to T2 (*p* = 0.028), T2 to T3 (*p* = 0.028), and T1 to T3 (*p* = 0.006), reflecting increased procedural efficiency with repeated practice.

When operative time was analyzed per incision method (Table 1), the reductions were driven by the laser settings with the longest initial times. Significant decreases from T1 to T3 were observed for the 1 W pulsed setting (87.9 vs. 50.0 s; Holm-adjusted *p* = 0.003) and the 3 W pulsed setting (39.8 vs. 23.3 s; *p* = 0.013). The faster settings were 1 W continuous (41.8 vs. 30.9 s; *p* = 0.152), 3 W continuous (22.2 vs. 18.2 s; *p* = 0.179), and the scalpel (23.5 vs. 17.5 s; *p* = 0.266) and showed numerical but non-significant reductions.

**Table 1:** Operative time by incision method and total session time (*n* = 22), in seconds. Means (SD) per time point; p = Holm-adjusted Wilcoxon signed-rank comparison T1 vs. T3. Bold = *p* < 0.05

Method	T1, mean (SD)	T2, mean (SD)	T3, mean (SD)	*p* (T1 vs. T3)
Laser, 1 W, pulsed	87.9 (65.7)	65.9 (30.6)	50.0 (26.3)	**0.003**
Laser, 3 W, pulsed	39.8 (30.7)	29.7 (18.3)	23.3 (11.8)	**0.013**
Laser, 1 W, continuous	41.8 (28.7)	37.8 (17.5)	30.9 (14.1)	0.152
Laser, 3 W, continuous	22.2 (12.2)	20.9 (13.6)	18.2 (9.9)	0.179
Scalpel	23.5 (12.1)	18.2 (8.7)	17.5 (10.5)	0.266
Total	215.3 (119.8)	172.6 (74.5)	139.8 (62.5)	**0.006**

### Subjective workload (NASA-TLX)

Perceived workload as measured by the NASA-TLX decreased substantially over the course of the study. Significant reductions were observed in the subscales mental demand (T1: mean 8.00, SD 3.80; T3: mean 4.77, SD 3.50; *p* < 0.001), physical demand (T1: mean 6.18, SD 3.96; T3: mean 3.82, SD 2.61; *p* = 0.013), goal achievement (T1: mean 11.9, SD 4.58; T3: mean 6.23, SD 3.78; *p* < 0.001), and frustration (T1: mean 6.95, SD 5.19; T3: mean 3.86, SD 3.08; *p* = 0.012). No significant changes were observed for temporal demand (*p* = 0.764) or effort (*p* = 0.068).

### Incision length

All four laser settings, as well as the scalpel cut, showed a significant improvement in incision length accuracy from T1 to T3, with the mean values for the absolute deviation from the 10 mm target decreasing accordingly (Table 2). For the 1 W pulsed setting, mean deviation incision length decreased from 2.47 mm (SD 1.79) at T1 to 1.03 mm (SD 0.59) at T3 (*p* = 0.011). Comparable improvements were observed for 3 W pulsed (T1: 2.38 mm, SD 1.77; T3: 1.01 mm, SD 0.81; *p* = 0.012), 1 W continuous (T1: 2.40 mm, SD 1.62; T3: 1.28 mm, SD 0.96; *p* = 0.026), and 3 W continuous (T1: 2.25 mm, SD 1.73; T3: 1.06 mm, SD 0.89; *p* = 0.026), the scalpel (T1: 2.35 mm, SD 1.50; T3: 1.30 mm, SD 1.05; *p* = 0.017).

**Table 2:** Incision dimensions (length, depth, width) by method and time point (T1–T3). Values are given as mean (SD) in mm (*n* = 22). P-values refer to the paired comparison T1 vs. T3; values in bold indicate statistical significance (*p* < 0.05). Target values were 10 mm (length), 2 mm (depth), and minimal width

Method	T1 mean (SD)	T2 mean (SD)	*T3 mean* (SD)	Δ T1→T3 (95% CI)	dz	*p*
Length: error from 10 mm						
Laser 1 W pulsed	2.47 (1.79)	2.00 (1.00)	1.03 (0.59)	−1.44 (− 2.30, − 0.58)	−0.74	**0.011**
Laser 3 W pulsed	2.38 (1.77)	1.68 (1.02)	1.01 (0.81)	−1.37 (− 2.22, − 0.52)	−0.72	**0.012**
Laser 1 W continuous	2.40 (1.62)	1.47 (1.08)	1.28 (0.96)	−1.12 (− 1.98, − 0.27)	−0.58	**0.026**
Laser 3 W continuous	2.25 (1.73)	1.19 (0.84)	1.06 (0.89)	−1.19 (− 2.10, − 0.28)	−0.58	**0.026**
Scalpel	2.35 (1.50)	1.92 (1.43)	1.30 (1.05)	−1.05 (− 1.76, − 0.34)	−0.66	**0.017**
Depth: error from 2 mm						
Laser 1 W pulsed	1.43 (0.16)	1.45 (0.14)	1.20 (0.26)	−0.23 (− 0.36, − 0.10)	−0.76	**0.009**
Laser 3 W pulsed	1.11 (0.47)	1.03 (0.39)	0.97 (0.31)	−0.14 (− 0.39, 0.10)	−0.26	0.578
Laser 1 W continuous	1.15 (0.36)	1.23 (0.28)	1.01 (0.33)	−0.14 (− 0.34, 0.05)	−0.32	0.578
Laser 3 W continuous	0.94 (0.49)	0.91 (0.40)	0.78 (0.34)	−0.16 (− 0.38, 0.07)	−0.31	0.578
Scalpel	0.70 (0.58)	0.81 (0.49)	0.60 (0.38)	−0.10 (− 0.37, 0.17)	−0.16	0.578
Width: raw (mm), target minimal						
Laser 1 W pulsed	0.91 (0.34)	0.99 (0.32)	0.88 (0.27)	−0.04 (− 0.21, 0.14)	−0.09	0.670
Laser 3 W pulsed	0.94 (0.25)	0.91 (0.27)	0.88 (0.21)	−0.07 (− 0.17, 0.04)	−0.28	0.633
Laser 1 W continuous	0.97 (0.27)	0.91 (0.24)	0.88 (0.24)	−0.09 (− 0.24, 0.06)	−0.26	0.633
Laser 3 W continuous	1.03 (0.23)	1.02 (0.24)	1.14 (0.25)	0.11 (− 0.02, 0.24)	0.38	0.408
Scalpel	0.67 (0.30)	0.55 (0.20)	0.79 (0.26)	0.12 (− 0.02, 0.26)	0.39	0.408

In the repeated-measures ANOVA (Table 3) of absolute length error, there was a significant main effect of time (F(2,42) = 11.6, *p* < 0.001; generalized η² = 0.14), no significant main effect of method (F(4,84) = 1.06, *p* = 0.37), and no significant method-by-time interaction (F(8,168) = 0.89, *p* = 0.44). Length accuracy therefore improved over the training period, and the magnitude of improvement did not differ significantly between the four laser settings and the scalpel.

**Table 3:** Two-way repeated-measures ANOVA results (method, time, method-time) for each dimension. F-statistics with numerator/denominator degrees of freedom, Greenhouse-Geisser–corrected p-values, and generalized η² (η²G). For length and depth, the ANOVA was computed on the target-based absolute error; for width, on the raw value. Bold = *p* < 0.05

Outcome	Effect	F (df1, df2)	*p*	η²G
Length accuracy	Method	1.06 (4, 84)	0.370	0.011
	Time	11.63 (2, 42)	**< 0.001**	0.144
	Method - Time	0.89 (8, 168)	0.444	0.013
Depth accuracy	Method	36.14 (4, 84)	**< 0.001**	0.268
	Time	5.25 (2, 42)	**0.010**	0.042
	Method - Time	0.66 (8, 168)	0.600	0.009
Width (raw)	Method	16.52 (4, 84)	**< 0.001**	0.195
	Time	0.51 (2, 42)	0.600	0.004
	Method - Time	3.49 (8, 168)	**0.011**	0.046

### Incision depth

Incision depth accuracy improved across all methods from T1 to T3, though statistical significance was reached only for the 1 W pulsed laser setting (T1: 1.43 mm, SD 0.16; T3: 1.20 mm, SD 0.26; *p* = 0.009). For the remaining laser settings and the scalpel, the trend towards the 2 mm target was consistent but did not reach the significance threshold. Among all methods at T3, the scalpel produced the closest approximation to the target depth, followed by the 3 W continuous laser setting (Table 2).

In the repeated-measures ANOVA (Table 3) of absolute depth error, there was a strong main effect of method (F(4,84) = 36.1, *p* < 0.001; generalised η² = 0.27), reflecting the systematically shallower laser incisions relative to the scalpel, a small but significant main effect of time (F(2,42) = 5.25, *p* = 0.010; generalised η² = 0.04), and no significant method-by-time interaction (F(8,168) = 0.66, *p* = 0.60).

### Incision width

Incision width, with the target to keep it as narrow as possible, remained largely stable across sessions for all laser settings (Table 2). Marginal improvements (i.e., reductions in width) were observed for 1 W pulsed (T1: 0.91 mm, SD 0.34; T3: 0.88 mm, SD 0.27; *p* = 0.670), 3 W pulsed (T1: 0.94 mm, SD 0.25; T3: 0.88 mm, SD 0.21; *p* = 0.633), and 1 W continuous (T1: 0.97 mm, SD 0.27; T3: 0.88 mm, SD 0.24; *p* = 0.633), none of which were statistically significant. The 3 W continuous setting showed a slight non-significant increase in width over time (T1: 1.03 mm, SD 0.23; T3: 1.14 mm, SD 0.25; *p* = 0.408). Laser incisions were consistently wider than scalpel incisions at T1 and T2 (large effect sizes, all *p* ≤ 0.001). At T3, the between-group difference no longer reached significance for three of the four laser settings. The 3 W continuous setting remained significantly wider than the scalpel (*p* < 0.001, Cohen’s d = 1.37).

In the repeated-measures ANOVA of incision width, there was a strong main effect of method (F(4,84) = 16.5, *p* < 0.001; generalized η² = 0.20), no significant main effect of time (F(2,42) = 0.51, *p* = 0.60), and a significant method-by-time interaction (F(8,168) = 3.49, *p* = 0.011).

### Histological findings

**Fig. 2:**
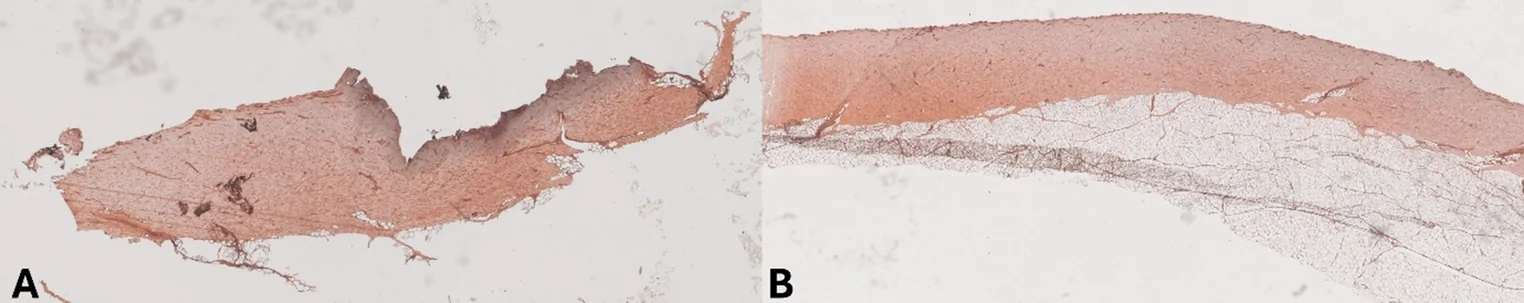
Representative HE-stained histological sections of the diode laser (**A**) and the scalpel (**B**) laser incisions show a central incision channel bordered by thermally altered marginal zones (coagulation and focal carbonization), whereas scalpel incisions display sharply demarcated margins without thermal alteration. The images are illustrative

HE-stained sections of laser-incised specimens revealed thermal alterations at the incision margins (Fig. 2). Thermally affected areas showed loss of distinct nuclear staining consistent with coagulation necrosis, increased eosinophilia of cytoplasmic and extracellular-matrix components, and focal carbonization appearing as dark brown to black areas under light microscopy. These marginal zones were distinguishable from adjacent intact tissue. Scalpel incisions showed sharply demarcated cut edges without thermal alteration. As the histological assessment was qualitative and descriptive, and no quantitative measurement of thermal damage was undertaken, these observations support the presence of thermally altered margins after laser incision and their absence after scalpel incision, but they do not support comparative claims regarding the extent of thermal damage among the individual laser settings.

## Discussion

This study investigated a structured preclinical diode-laser training program for undergraduate dental students without prior laser experience, using an ex vivo porcine skin model, and evaluated performance in terms of target-based accuracy, operative time, and subjective workload. The main finding was an early learning effect in operative efficiency, perceived workload, and length accuracy. Importantly, the improvement in length control was comparable across all incision conditions, including the familiar scalpel, and there was no method-by-time interaction. This indicates that the observed gains largely reflect general task familiarisation rather than a laser-specific learning advantage.

The significant reduction in total operative time from a mean of 215 s at T1 to 140 s at T3, together with the per-method analysis, demonstrates that novice operators can rapidly acquire basic procedural efficiency with the diode laser. The per-method data add nuance: the largest and only statistically significant reductions occurred for the two pulsed laser settings, which were the slowest and least familiar at baseline, whereas the already-fast scalpel condition did not change significantly. This finding is consistent with learning curve analyses in other surgical domains, where the most substantial efficiency gains typically occur in the early phase of skill acquisition [9, 15]. Surgical procedures requiring a certain aptitude for technical tasks can be learned, but they demand specific training and must be repeated multiple times to attain a certain level of surgical experience [16]. Experience appears to be of particular importance, especially in oropharyngeal laser tumor surgery, to keep mortality and morbidity as low as possible [17]. Structured instruction and assistance for residents can also be important in improving complication management during ophthalmic laser surgery [18]. Kosiba et al. demonstrated similar results for laser-assisted prostate surgery, showing that a structured mentoring program ensures safety and efficacy during the learning curve while maintaining excellent outcome quality [19]. Ceviz et al. confirm that surgical training using models, ideally within the framework of mentoring programs, can improve the success rates of initial operations and reduce the mental strain associated with surgical specialties [20].

This is also consistent with our study, as four of the six NASA-TLX metrics improved significantly. Practicing laser surgical procedures on a model beforehand could reduce surgeons’ perceived workload during initial oral applications. In particular, the marked decrease in the goal achievement subscale, reflecting the gap between desired and actual performance, suggests that students developed increasing confidence in their ability to meet the procedural targets. Süer et al. recognized that integrating the NASA-TLX can help visualize real-time workload during laparoscopic procedure simulation and training, thereby improving patient safety in the long term [21]. Lund et al. reported that high workload can result in negative NASA-TLX scores among both junior and experienced physicians, a finding that may be associated with burnout and shift performance [22]. Thus, recording data, even during practice on a model, makes sense, as it allows trainees to see not only their learning curve but also their reduced perceived workload.

On the target-based scale, absolute length error decreased significantly and comparably for all conditions, including the familiar scalpel, and the absence of a method-by-time interaction indicates that these gains reflect general task familiarisation rather than a laser-specific learning advantage. Depth was the least well-mastered dimension, remaining well below the 2 mm target and improving for only a single laser setting, most likely because novice operators worked cautiously on the ex vivo specimens, the non-contact laser lacks the tactile feedback of a scalpel, and the visual endpoint encouraged termination once a groove was visible rather than at a defined depth. These morphological findings are consistent with the descriptive histology, which showed thermal margins after laser but not scalpel incision while supporting no quantitative ranking among laser settings, and together they identify depth control and limitation of lateral thermal spread as the principal targets for extended training.

Beyond statistical significance, the clinical relevance of these findings should be considered. The diode laser can be an effective tool in oral soft-tissue surgery, offering reduced bleeding, improved intraoperative visibility, reduced pain perception, and potentially shorter operative time [23, 24]. However, for these benefits to be realized, a targeted surgical procedure must be performed to ensure that the incision length, width, and depth are precisely maintained. The significant improvement in incision length across all laser settings in this study confirms this statement. The absence of significant improvements in incision depth across most settings underscores the greater complexity of three-dimensional incision control compared to two-dimensional path guidance. Depth regulation with a laser requires the operator to modulate hand speed and tissue dwell time in real time, a skill that appears to require more extensive practice than acquiring an appropriate path length. Diode lasers are frequently and successfully used for gingivectomy in oral surgery. The advantages of minimal bleeding are highlighted in this context. Precise cutting is essential for this procedure [25]. Ultimately, however, the benefits of various lasers are critically discussed in the literature, particularly regarding periodontal surgery. A decisive factor in this regard is the lack of standardized parameters [26]. This aligns with the statement by Dalvi et al., who call for the laser to be used during patient treatment only by trained and experienced physicians [27]. However, this could be improved by integrating laser surgery into student training, so the initial application would not be performed directly on the patient.

Unlike scalpel blades, which produce mechanically defined cuts with minimal tissue displacement, laser incisions involve thermal ablation and vaporization together with a surrounding zone of coagulation that inherently contributes to incision width. Accordingly, laser incisions remained consistently wider than scalpel incisions, and no method showed a significant change in width across sessions. The narrowing of the laser-versus-scalpel difference at the final time point should not be interpreted as convergence or equivalence, as a failure to reject the null hypothesis in a small sample without a prespecified equivalence margin does not establish it. Notably, the 3 W continuous setting remained significantly wider than the scalpel throughout. The slight increase in width observed for this setting may reflect greater cumulative heat input at higher power in continuous mode. This raises the hypothesis, which would require confirmation by quantitative histomorphometry, that low-power or pulsed delivery might reduce lateral thermal spread. Because the histological assessment was purely descriptive, it cannot rank the individual settings. Qualitatively, however, the coagulation and focal carbonization observed at the laser-incision margins are consistent with the established zones of laser-tissue interaction. Every practitioner should therefore assess for which procedure and at which settings the diode laser represents a suitable alternative for a given oral surgical indication [28].

The present study has several limitations that should be considered when interpreting the findings. First, this is a single-center study conducted at a single institution, which may limit its generalizability. Second, the ex vivo porcine skin model, while histologically and biomechanically similar to human skin in many respects, does not fully replicate intraoral anatomical conditions, including keratinization, thickness, moisture, vascularity, mechanical constraints, the presence of saliva, or bleeding. Whether these thermal marginal zones affect clinical healing outcomes in oral mucosa warrants further investigation in in vivo models. Transferability of the findings to clinical intraoral conditions is therefore limited. Third, the study included only three training sessions; a longer follow-up would be necessary to determine the trajectory of skill consolidation and whether the observed improvements in depth and width control eventually reach significance with extended practice. Fourth, the micro-CT measurement approach used method-specific curve fitting and was not formally validated. Intra- and inter-rater reliability were not assessed, and no comparison against a physical reference standard was performed, so the method is described as feasible and potentially objective rather than validated. Fifth, the scalpel was a clinically familiar reference condition rather than an experience-matched comparator, so between-technique differences in learning must be interpreted with this imbalance in mind. Moreover, the five incision conditions were performed in a fixed order, which exposes the study to potential order effects, within-session learning, fatigue, and progressive changes in tissue handling. Future experiments should randomize or counterbalance the sequence, for example using a Latin-square design.

## Conclusions

Within the constraints of a single-center feasibility study, structured preclinical diode-laser training on an ex vivo porcine-skin model was feasible and was associated with early improvements in operative efficiency, perceived workload, and target-based length accuracy in undergraduate dental students without prior laser experience. Because comparable gains in length accuracy were observed for the familiar scalpel and no method-by-time interaction was found, these improvements are best attributed to general task familiarisation rather than a laser-specific learning advantage. Control of incision depth and width remained inconsistent, identifying three-dimensional depth control and limitation of lateral thermal spread as the principal targets for extended, feedback-rich training. The combination of a porcine-skin model with micro-CT analysis is a promising and feasible approach for objectively characterizing incision quality in a training context. These findings support the further development and evaluation of structured laser training within undergraduate dental education, but they cannot establish clinical competence, equivalence to scalpel surgery, or transfer of skills to intraoral patient care; randomized, adequately powered, and in vivo studies are warranted to confirm these preliminary results.

## Data Availability

No datasets were generated or analysed during the current study.
